# The Limonoids TS3 and Rubescin E Induce Apoptosis in Human Hepatoma Cell Lines and Interfere with NF-κB Signaling

**DOI:** 10.1371/journal.pone.0160843

**Published:** 2016-08-12

**Authors:** Nicole Lange, Armelle Tsamo Tontsa, Claudia Wegscheid, Pierre Mkounga, Augustin Ephrem Nkengfack, Christine Loscher, Gabriele Sass, Gisa Tiegs

**Affiliations:** 1 Institute of Experimental Immunology and Hepatology, University Medical Center Hamburg Eppendorf, Hamburg, Germany; 2 University of Yaoundé I, Department of Organic Chemistry, P.O BOX: 812, Yaoundé, Cameroon; 3 California Institute for Medical Research, San Jose, CA, United States of America; University of Navarra School of Medicine and Center for Applied Medical Research (CIMA), SPAIN

## Abstract

Hepatocellular carcinoma (HCC) is extremely resistant towards pharmacological therapy. To date, the multi-kinase inhibitor Sorafenib is the only available therapeutic agent with the potential to prolong patient survival. Using the human hepatoma cell lines HepG2 and Huh7, we analyzed anti-cancer activities of 6 purified havanensin type limonoids isolated from the traditional African medicinal plant *Trichilia rubescens* Oliv. Our results show that two of the compounds, TR4 (TS3) and TR9 (Rubescin E) reduced hepatoma cell viability, but not primary hepatocyte viability, at TC50s of 5 to 10 μM. These were significantly lower than the TC50s for Sorafenib, the histone deacetylase inhibitor SAHA or 5-Fluoruracil. In comparison, TR3 (Rubescin D), a limonoid isolated in parallel and structurally highly similar to TR4 and TR9, did not interfere with hepatoma cell viability. Both, TR4 and TR9, but not TR3, induced apoptosis in hepatoma cells and interfered with NF-κB activation. TR4 as well as TR9 significantly supported anti-cancer activities of Sorafenib. In summary, the limonoids TR4 and TR9 exhibit anti-cancer activities and support Sorafenib effects *in vitro*, having the potential to support future HCC therapy.

## Introduction

Hepatocellular carcinoma (HCC) is one of the most frequent causes of death from cancer, with more than 500.000 annual deaths [1], with an average survival time of one year following diagnosis [2]. Resection is possible in less than 30% of the cases, prolonging survival time over 5 years from 20 to 40% [3–5]. Clinical studies encompassing cryo-, radio-, hormone-, immuno- or chemotherapy show inconsistent and dissatisfactory results with respect to HCC regression as well as patient survival [6]. So far, solely the multi-kinase inhibitor sorafenib has been shown to prolong patient survival for an average of 4 to 8 months [7, 8].

These facts are a strong motivation to discover new anti-cancer substances or find support for existing therapy. A source of those new substances might be found in traditional medicine. Chinese, but with increasing frequency also African traditional medical plants are investigated for effects on various diseases [9, 10]. Among anti-cancer substances derived from those plants, predominantly flavonoids have been described [11, 12]. Limonoids have been shown to interfere with viability of colon carcinoma [13–15] and breast cancer cell lines [16–18]. Recently the limonoid nimbolide has been shown to induce apoptosis in human hepatoma cells at a TC50 of 5 μM [19].

The limonoids investigated here were isolated from *Trichilia rubescens* (Meliaceae), which encompasses about 90 species of mostly evergreen trees, widely distributed in tropical and subtropical regions [20]. Thirteen species of the genus *Trichilia* are growing in Cameroon [21], e.g. *Trichilia rubescens*. Leaves, roots and stem bark of this plant are used in traditional medicine [22]. Limonoids are a class of secondary metabolites. These polyoxygenated tetranortriterpenoids are derived from tirucallane (20S) or euphane (20R) with a β-substituted furanyl ring at C-17α [21, 23, 24]. Previously, the havanensin-type limonoids TS1, TS2, TS3 [25], trichirubines A and B [26] and rubescins A, B and C [27] have been isolated from *T*. *rubescens*. Some of these substances have been shown to increase chloride conductance in epithelial cells [25], and to exhibit anti-plasmodial [26], and antioxidant [27] activities.

Here we analyzed the anti-cancer activity of 6 limonoids isolated from organic extracts of the roots and stem bark of *T*. *rubescens* [25, 27, 28]. Our results show that 2 of these limonoids, TR4 (TS3) and TR9 (Rubescin E), interfered with human hepatoma cell viability at lower TC50s than Sorafenib, and support its anti-cancer activity. Apoptosis induction in hepatoma cells by TR4 and TR9 seems to be due to interference with NF-κB activation.

## Materials and Methods

### Ethics Statement

All animal experiments were performed according to local regulations of the state authorities in Hamburg, Germany. In particular, permission was obtained from ‘‘Amt für Gesundheits- und Verbraucherschutz” of ‘‘Behörde für Soziales, Familie, Gesundheit und Verbraucherschutz”, State Government of Freie und Hansestadt Hamburg, for perfusion of the liver and removal of hepatocytes from anaesthetized mice (registration number G21301/591-00.33, obtained on September 1, 2009). Regarding field studies, the University of Cameroon in Yaoundé states that *Trichilia rubescens* is not an endangered or protected species in Cameroon. It does not belong to the species under CITES (The Convention on International Trade in endangered species of wild fauna and flora) regulation. Subsequently, and according to Cameroonian regulation, no permission is required to collect and study this plant, especially in the Universities and Research Center Laboratories.

### Plant Material

The root and stem barks of *Trichilia rubescens* were collected in Mendong (Eloudem), Central region of Cameroon, in October 2011. A voucher specimen documenting the collection has been deposited at the National Herbarium of Cameroon under N° 38705/SRF Cam.

### Extraction and isolation

Air dried and powdered stem (1 kg) and root bark (1 kg) of *Trichilia rubescens* was extracted by maceration during 2 days at room temperature with a mixture of methanol (MeOH) and methylene chloride (CH_2_Cl_2_) (1:1). Following filtration and removal of the solvent under vacuum, 60 g and 150 g of dark greenish extracts were obtained, respectively. Fifty grams of stem bark extract were subjected to column chromatography, which was performed on silica gel and eluted with a hexane/EtOAc gradient of increasing polarity from hexane/ ethyl acetat (EtOAc) (9:1) to hexane/EtOAc (1:1), resulting in the isolation of compound TR3 (Table 1) using hexane/EtOAc (11:9) as the eluting solvent.

**Table 1:** Limonoids used in this study.

Name used in study	Trivial name	Reference
TR3	Rubescin D	[28]
TR4	TS3	[25]
TR8	Rubescin B	[27]
TR9	Rubescin E	[28]
TR11	TS1	[25]
TR12	Rubescin C	[27]

One hundred forty grams of the root bark extract were subjected to flash silica gel chromatography with hexane/EtOAc gradient of increasing polarity to obtain 4 fractions (F_1,_ F_2_, F_3_, and F_4_) monitored by thin-layer chromatography (TLC). Fraction F_2_ was subjected to silica gel column using a gradient mixture of hexane/EtOAc (20:1 to 5:1) to yield 100 fractions of 125 mL each. Based on TLC, fractions with similar compounds were combined, resulting in series 1–8 (S_1_ (1–29), S_2_ (30–35), S_3_ (39–41), S_4_ (46–53), S_5_ (63–66), S_6_ (68–72), S_7_ (84–87) and S_8_ (90–100)). Crystallization and filtration of S_2_ resulted in the isolation of compound TR7. S_3_ was subjected to silica gel column chromatography using hexane/EtOAc (4:1), which resulted in the isolation of compound TR8 (Table 1). S_4_ was chromatographed on a silica gel column and eluted successively with hexane/EtOAc (3:1) to obtain compound TR9 (Table 1). In a similar procedure S_6_ was crystalized and filtered to obtain compound TR11 (Table 1). Series S_7_ was subjected to column chromatography (70–230 mesh, 1.5 x 90 cm column) with gradient of increasing polarity from hexane to hexane/EtOAc (1:1), to obtain compound TR12 (Table 1) using hexane/EtOAc (13:7) as an eluent.

Fraction F_3_, eluted with hexane, was further chromatographed on silica gel and eluted with a gradient of increasing polarity from hexane to hexane/EtOAc (1:1) to give 180 fractions of 125 mL each, which were combined on the basis of their TLC profiles to produce 9 main fractions A_1_-A_9._ Of these, fraction A_7_ was crystalized and filtered, resulting in compound TR4 (Table 1) (100 mg) using hexane/EtOAc (3:2) as the eluting solvent.

### Experimental Procedures for Structure Determination

Optical rotations were recorded on a Perkin-Elmer Model 2000 polarimeter. Melting points were determined on a Buchii melting point apparatus and are uncorrected. IR spectra were recorded on Bruker Fourier transform/infrared (ATR) spectrophotometer. ^1^H and ^13^C nuclear magnetic resonance spectroscopy (NMR) was run on a Bruker AV-300 and -500 spectrometer equipped with 5 mm ^1^H (300 MHz and 500 MHz) and ^13^C (75 MHz and 125 MHz) probes operating at 300 and 75 MHz and 500 and 125 MHz, respectively either in deuterated chloroform (CDCl_3_), deuterated methanol (MeOD) or deuterated pyridine (C_5_D_5_N) with Tetramethylsilane (TMS) as internal standard. High resolution mass spectrometry (Electrospray Ionization and Electronic Impact) were run on a Varian mass spectrometer (Varian Inc., Palo Alto-California). Silica gels (Merck, 230–400 and 70–230 mesh) were used for flash and column chromatography. TLC analyses were performed on silica gel 60F254 precoated alumina sheets (0.2 mm layer thickness). Spots were visualized under UV lamp (254 nm and 365 nm) or by heating after spraying with 10% H_2_SO_4_ reagent. Different mixtures of hexane, EtOAc, CH_2_Cl_2_ and MeOH were used as eluent solvents. Individual limonoids were dissolved in dimethyl sulfoxide (DMSO) with stock concentrations of 10 mM. Ten μl aliquots were stored at -20°C.

#### Reagents

Sorafenib (LC Laboratories, Woburn, USA); Suberoylanilide hydroxamic acid (SAHA; kind gift of Prof. Matthias Ocker, University of Marburg); 5-Fluorouracil (5-FU; Sigma Aldrich GmbH, Steinheim, Germany); Actinomycin D (ActD; Th. Geyer Hamburg GmbH & Co. KG, Hamburg, Germany); recombinant mouse TNFα (TNFα; SINO Biological Inc., Beijing, China).

#### Cell culture

For experiments, all limonoids were dissolved in DMSO at stock concentrations of 10 mM. Dilutions were performed in the cell culture medium used for the respective experiments, as described below. In each experiment a control was implemented, containing the same or highest DMSO content used. The human hepatoma cell lines Huh7 [29] and HepG2 [30] were cultured in DMEM+GlutaMAX™-I containing 10% fetal calf serum (FCS) and penicillin [100U/ml] / streptomycin [100 μg/ml] (Merck KGaA-Biochrom AG, Berlin, Germany). The mouse hepatoma cell line Hepa1-6 [31] was maintained in RPMI-1640+GlutaMAX™-I medium containing 10% FCS and penicillin [100U/ml] / streptomycin [100 μg/ml]. Primary mouse hepatocytes were prepared from 10–11 weeks old male C57Bl/6 mice as described previously and cultured in William´s E+GlutaMAX™-I medium containing 10% FCS penicillin [100U/ml] / streptomycin [100 μg/ml] and L-glutamine [c = 2 mM]) as described previously [32, 33]. All cell culture media, L-glutamine and FCS are obtained from Life Technologies GmbH-GIBCO (Darmstadt, Germany). For experimental procedures, cells were seeded into 6-, 24- or 96-well plates and allowed to adhere overnight. The next day cells were incubated as indicated.

#### Analysis of cell viability

Cell viability was measured by using (3–4, 5-Dimethylthiazol-2-yl)-2, 5-diphenyltetrazolium bromide (MTT; Sigma Aldrich GmbH, Steinheim, Germany) according to the manufacturer’s instructions.

#### Detection of mRNA by RT-PCR

To verify altered gene expression, 1 μg RNA was transcribed into cDNA using the Verso^TM^ cDNA Kit (Thermo Fisher Scientific, Waltham, USA). Oligonucleotides for subsequent PCR-reactions were obtained from Metabion International AG (Martinsried, Germany) and are summarized in Table 2. Real time RT-PCR was performed using the CFX^TM^ Real-Time system (BIO-RAD, Munich, Germany) and reagents from ABgene® (Epsom, UK). To confirm amplification specificity, PCR products were subjected to melting curve analysis and gel electrophoresis.

**Table 2:** qPCR sequences.

Target	Forward primer	Reverse primer	Reference
mATPsy	GCCCACTTCTTACCACAAGG	GCGACAGCGATTTCTAGGAT	AF368271
PCNA	GGCGTGAACCTCACCAGTAT	TCTCGGCATATACGTGCAAA	NM002592
HSA	AATGCCCTGTGCAGAAGACT	TCATCGACTTCCAGAGCTGA	AF542069
p21	GACTTTGTCACCGAGACACC	GACAGGTCCACATGGTCTTC	AF497972.1
Bcl-2	TGTGGCCTTCTTTGAGTTCG	GAGAAATCAAACAGAGGCCG	NM_000657.2
Bcl10	TCCTCTCCTTCTTCCCCATT	GGCGTCCTTCTTCACTTCAG	NM003921

#### DNA-fragmentation analysis

Cells were harvested and prepared as described [34]. Due to this method only fragmented DNA was isolated.

#### Protein isolation and Western Blot analysis

Whole cell lysate were prepared using 50 mM Tris/HCl (pH 8.0), 150 mM NaCl, 1% NP40, 0.5% Na-Deoxycholat, 5 mM EDTA, 0.1% SDS, 1 x Phosphatase Inhibitor Cocktail (Roche Applied Sience, Mannheim, Germany) and 1 x Protease Inhibitor Cocktail (Sigma-Aldrich, Steinheim, Germany). Separations of cytosolic and nuclear fractions were performed as described previously [34]. For Western Blot analysis 40 μg whole cell lysate or nuclear cell fraction were fractionated by 12% SDS-PAGE, blotted onto nitrocellulose membranes and incubated over night with the indicated antibodies. Western blots were developed using a self-made ECL buffer (1.25 mM Luminol/TrisHCl pH 8.6, 15 mM Parahydroxy-Coumarinacid/DMSO and 0.01% H_2_O_2_). Semiquantitative evaluations were performed using the VersaDoc^TM^ Imaging System 4000 MP (Bio-Rad, Munich, Germany) followed by quantification using the Image Lab^TM^ Software (Bio-Rad, Munich, Germany).

#### Antibodies

Goat anti-GAPDH (V-18) (Santa Cruz Biotechnology, Inc., Santa Cruz, USA), goat anti-Cyclin D1 (CCND1) (antibodies-online GmbH, Aachen, Germany), rabbit anti-IκBα (Epitomics, Abcam, Burlingame, USA), rabbit anti-Caspase-3 (8G10), rabbit anti-PARP (46D11), rabbit anti-phospho-IκBα (Ser32) (14D4), rabbit anti-A20/TNFAIP3 (D13H3) (all: Cell Signaling Technologies, New England Biolabs, Frankfurt am Main, Germany).

#### NF-κB Luciferase Reporter Assay

Cells were seeded in 24-well plates and transfected with 0.8 μg of NF-κB reporter vector pB2LUC, encoding firefly luciferase under the control of two NF-κB promoter elements, using Lipofectamin 2000 (Invitrogen, Darmstadt, Germany). For activation of the NF-κB signaling pathway cells were incubated with 40 ng/ml TNFα for 4 hours. Luciferase activity was measured using the Luciferase Assay System (Promega, Mannheim, Germany), and normalized to the protein content in the individual sample.

#### BrdU- and Caspase-3-Assay

Cell Proliferation was measured using a BrdU cell proliferation chemiluminescent assay kit (Cell Signaling Technologies, New England Biolabs, Frankfurt am Main, Germany). Apoptosis was determined using the Caspase-3 colorimetric assay kit (Sigma Aldrich GmbH, Steinheim, Germany). Both assays were performed according to the manufacturer’s protocols.

#### FACS analysis

Cells were cultured and stimulated as described above and harvested by trypsinisation. A total of 200.000 cells were stained using a standard Annexin V (Alexa Fluor 647) and Propidium Iodide (PI) staining protocol (all: BioLegend, Fell, Germany). Data were recorded and analyzed using the BD FACSCanto II system and BD FACSDiva software (BD Biosciences, Heidelberg, Germany).

#### Statistical analysis

Results were analyzed using Student’s *t* test, if two groups were compared and the Bonferroni’s test if more groups were tested against a control group. All data in this study are expressed as a mean ± SEM. ★ p ≤ 0.05; ★★ p ≤ 0.01; ★★★ p ≤ 0.001.

## Results

### The limonoids TR4 and TR9 have strong adverse effects on human hepatoma cell viability

**Fig 1:**
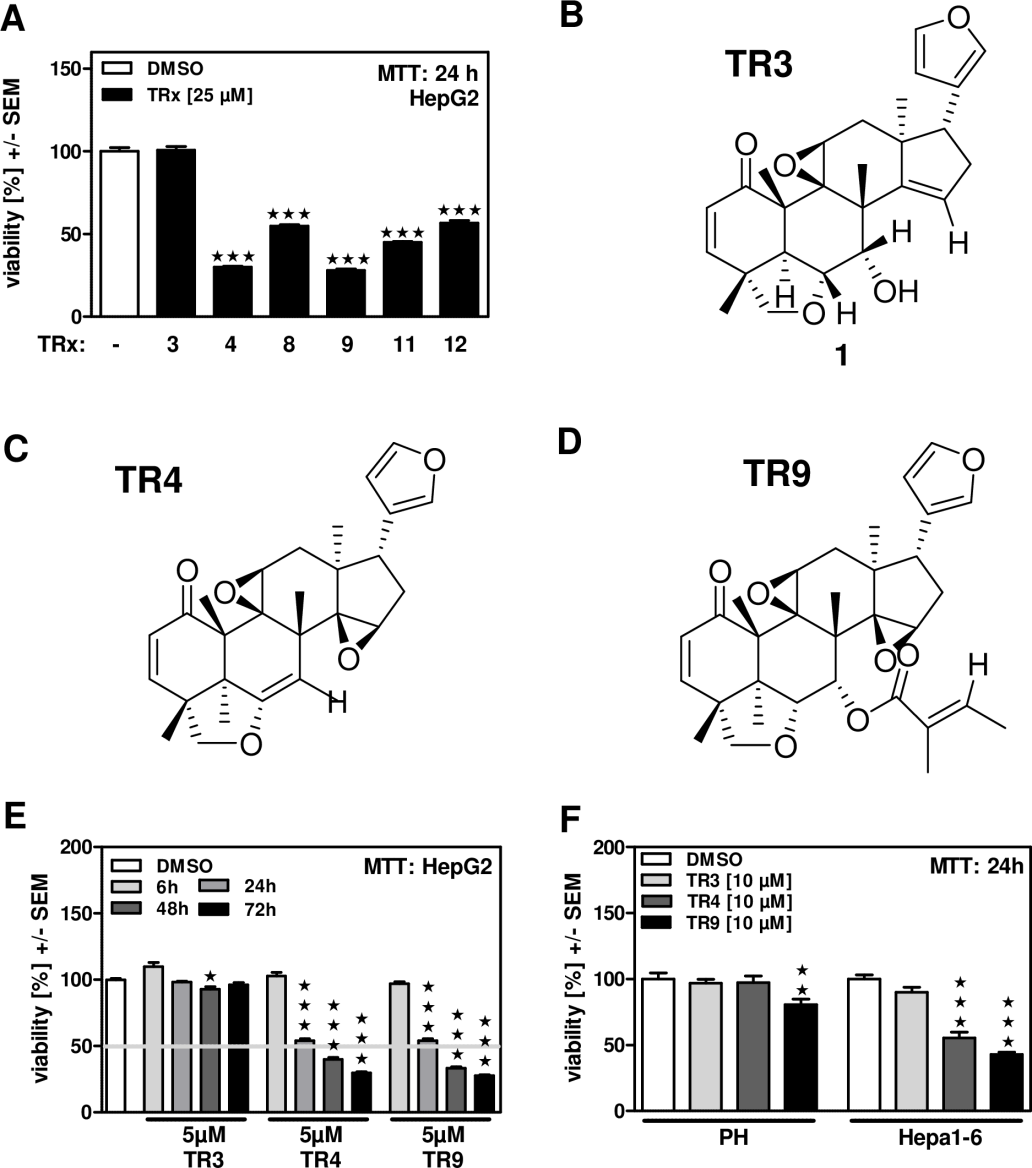
The limonoids TR4 and TR9 have strong adverse effects on human hepatoma cell viability. A: HepG2 human hepatoma cells were incubated with 25 μM of 6 different limonoids (TR3, TR4, TR8, TR9, TR11 and TR12, isolated from *Trichilia rubescens* Oliv.) for 24 hours. Cell viability was measured by MTT assay. DMSO control: 0.25%. B-D: Structures of the most powerful limonoids TR4 and TR9 as well as the structurally similar but ineffective control limonoid TR3. E: HepG2 cells were incubated with 5 μM of TR3, TR4 or TR9 for 6 to 72 hours. Cell viability was measured by MTT assay. The grey line indicates the TC50. DMSO control: 0.05%. F: Primary mouse hepatocytes (PH) isolated from male C57/Bl6 mice, as well as corresponding mouse hepatoma cells (Hepa1-6) were incubated with 10 μM TR3, TR4 or TR9 for 24 hours. Cell viability was measured by MTT assay. DMSO control: 0.1%. ★★ p ≤ 0.01, ★★★ p ≤ 0.001 vs DMSO controls.

We investigated the ability of six limonoids isolated from *Trichilia rubescens* Oliv. to interfere with HepG2 human hepatoma cell viability *in vitro*. Our results show that at concentrations of 25 μM five out of the six limonoids were able to significantly reduce HepG2 cell viability within 24 h (Fig 1A). While TR8, TR11 and TR12 reduced HepG2 cell viability by approximately 50%, TR4 and TR9 reduced cancer cell viability by about 70%. The limonoid TR3 did not have any effect on cell viability (Fig 1A). Chemical structures of TR3, TR4 and TR9 are shown in Fig 1B–1D. Further investigation revealed that TR4 and TR9 effects on cell viability increased dose-dependently over 72 h (Fig 1E). With regard to possible adverse effects on non-tumor cells in the liver we compared viability of TR3, TR4 or TR9 incubated mouse hepatoma cells (Hepa1-6) to viability of freshly isolated primary mouse hepatocytes (PH). Our results show that 10 μM of TR4 and TR9, but not TR3, significantly decreased viability of Hepa1-6 cells (Fig 1F). Neither TR3 nor TR4 interfered with the viability of PH, while TR9 had a significant effect. TR9 reduced the viability of PH by about 20%, while it decreased viability of the corresponding hepatoma cells by about 60%. In conclusion, TR4 and TR9 seem to have a much higher impact on hepatoma cells than on primary cells *in vitro*.

### TC50 values of TR4 and TR9 for human hepatoma cells are lower than those of Sorafenib, SAHA or 5-Fluorouracil (5-FU)

**Fig 2:**
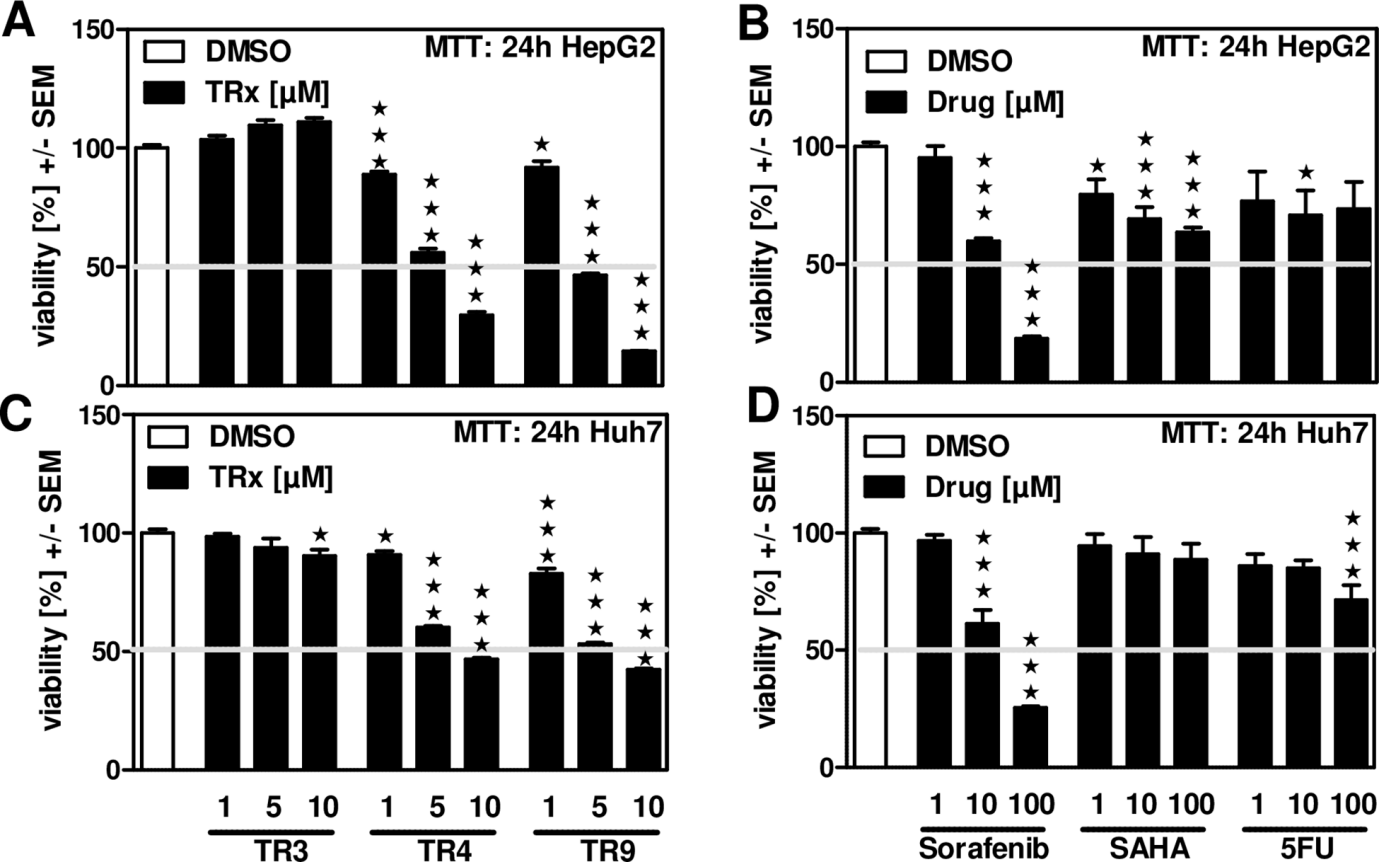
TR4 and TR9 decrease hepatoma cell viability at a lower TC50 than Sorafenib, SAHA or 5-Fluorouracil. HepG2 (A, B) or Huh7 (C, D) human hepatoma cells were incubated with 1, 5 or 10 μM TR3, TR4 or TR9 (A, C) or 1, 10 or 100 μM Sorafenib, SAHA or 5-Fluorouracil (5-FU) (B, D) for 24 hours. Cell viability was measured by MTT assay (A-D). Grey lines indicate TC50s. DMSO control: 0.1%. ★★ p ≤ 0.01, ★★★ p ≤ 0.001 vs DMSO controls.

Dose/response analysis for the most effective compounds, TR4 and TR9, revealed TC50 values of about 7 μM for TR4 in HepG2 cells (Fig 2A) and 10 μM in Huh7 cells (Fig 2C), while TC50 values of TR9 were about 5 μM in both, HepG2 and Huh7 cells (Fig 2A and 2C). Again TR3 did not show effects on cell viability (Fig 2A and 2C). In parallel we estimated TC50 values on human hepatoma cells for the multi-kinase inhibitor Sorafenib, the histone deacetylase inhibitor SAHA and the anti-metabolite 5-FU. Our results show a TC50 value for Sorafenib higher than 10 μM in HepG2 (Fig 2B) or Huh7 cells (Fig 2D), while neither SAHA nor 5-FU were able to inhibit hepatoma cell viability to the TC50 range at concentrations up to 100 μM (Fig 2B and 2D).

### TR4 and TR9 inhibit proliferation of human hepatoma cells

**Fig 3:**
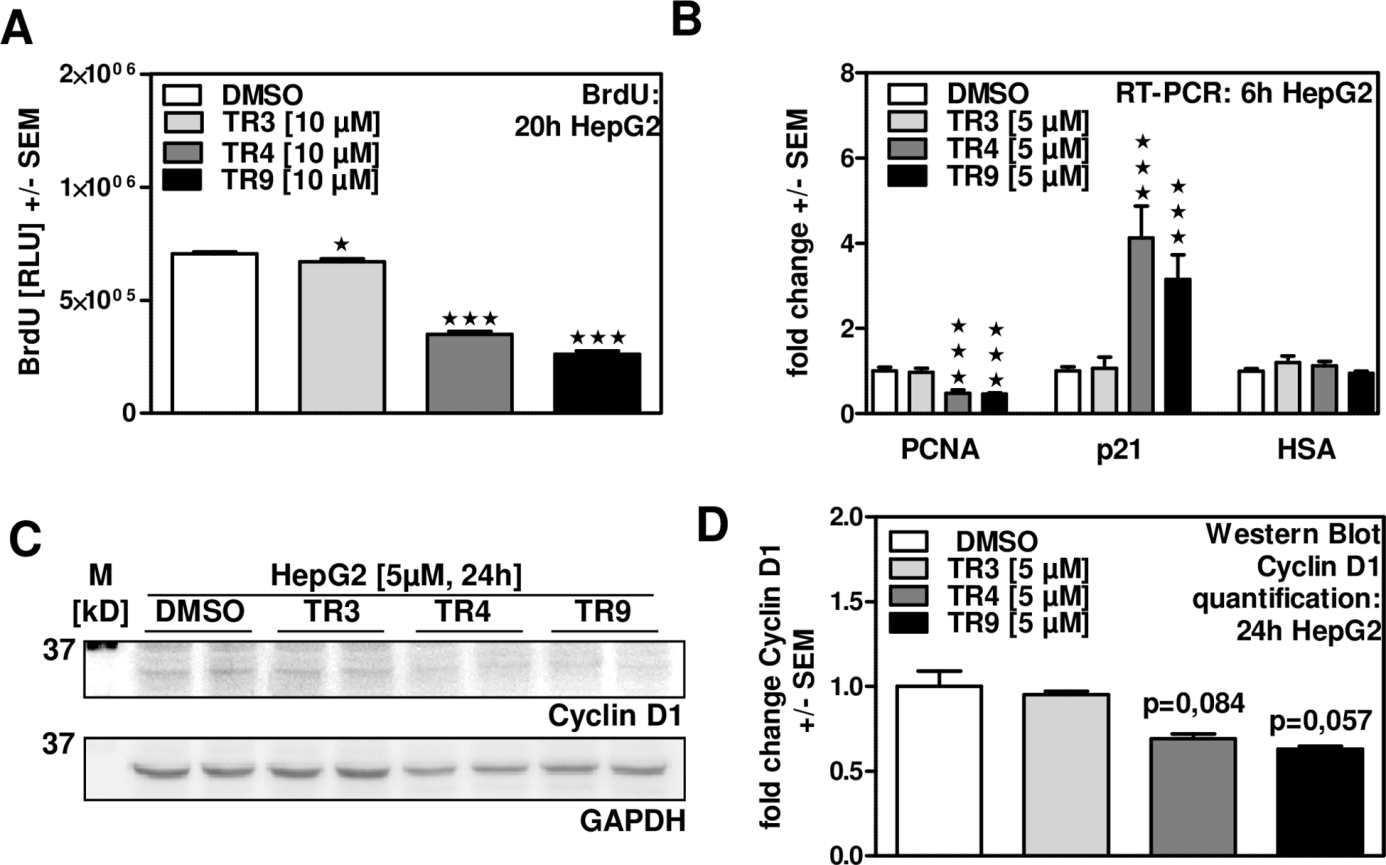
TR4 and TR9 inhibit proliferation of human hepatoma cells. A: HepG2 cells were incubated with 10 μM TR3, TR4 or TR9 for 20 hours. Cell proliferation was measured by BrdU-Assay. DMSO control: 0.1%. B: HepG2 cells were incubated with 5 μM TR3, TR4 or TR9 for 6 hours. Expression of PCNA, p21 and HSA was measured by real time RT-PCR. DMSO control: 0.05%. ★ p ≤ 0.05, ★★★ p ≤ 0.001 vs DMSO controls. C + D: HepG2 cells were incubated with 5 μM TR3, TR4 or TR9 for 24 hours. Protein expression of Cyclin D1 was detected by Western Blot analysis (C) and quantified by using Image Lab^TM^ Software (D). Quantification was performed in relation to reference expression of GAPDH as a loading control.

Since a loss in cell viability observed by MTT assay might be due to decreased cell proliferation or actual cell death, we next determined TR4 and TR9 effects on cell proliferation. Our results show that 10 μM of TR4 reduced HepG2 cells proliferation by about 50%, while 10 μM of TR9 reduced proliferation by about 60%. TR3 had only minor effects on HepG2 proliferation (about 5% decrease) (Fig 3A). In line with these results, we found that expression of PCNA, a cofactor of DNA replication, was decreased following TR4 or TR9 incubation, while HSA expression, as a control for metabolic activity, was unaffected. Additionally, we observed an increase in p21 expression, indicating promotion of cell-cycle arrest (Fig 3B). TR3 did not alter expression levels of PCNA, p21 or HSA (Fig 3B). Furthermore, we determined whether TR4 or TR9 affected protein expression of Cyclin D1, a protein involved in G1/S phase transition of the cell cycle. Our results show that protein levels of Cyclin D1 declined in the presence of TR4 and TR9, while TR3 did not affect Cyclin D1 expression within 24 h (Fig 3C). These results were quantified by using Image Lab^TM^ Software (Fig 3D). In conclusion, TR4 and TR9 exert inhibitory effects on proliferation of human hepatoma cells.

### TR4 and TR9 induce apoptosis in human hepatoma cells

**Fig 4:**
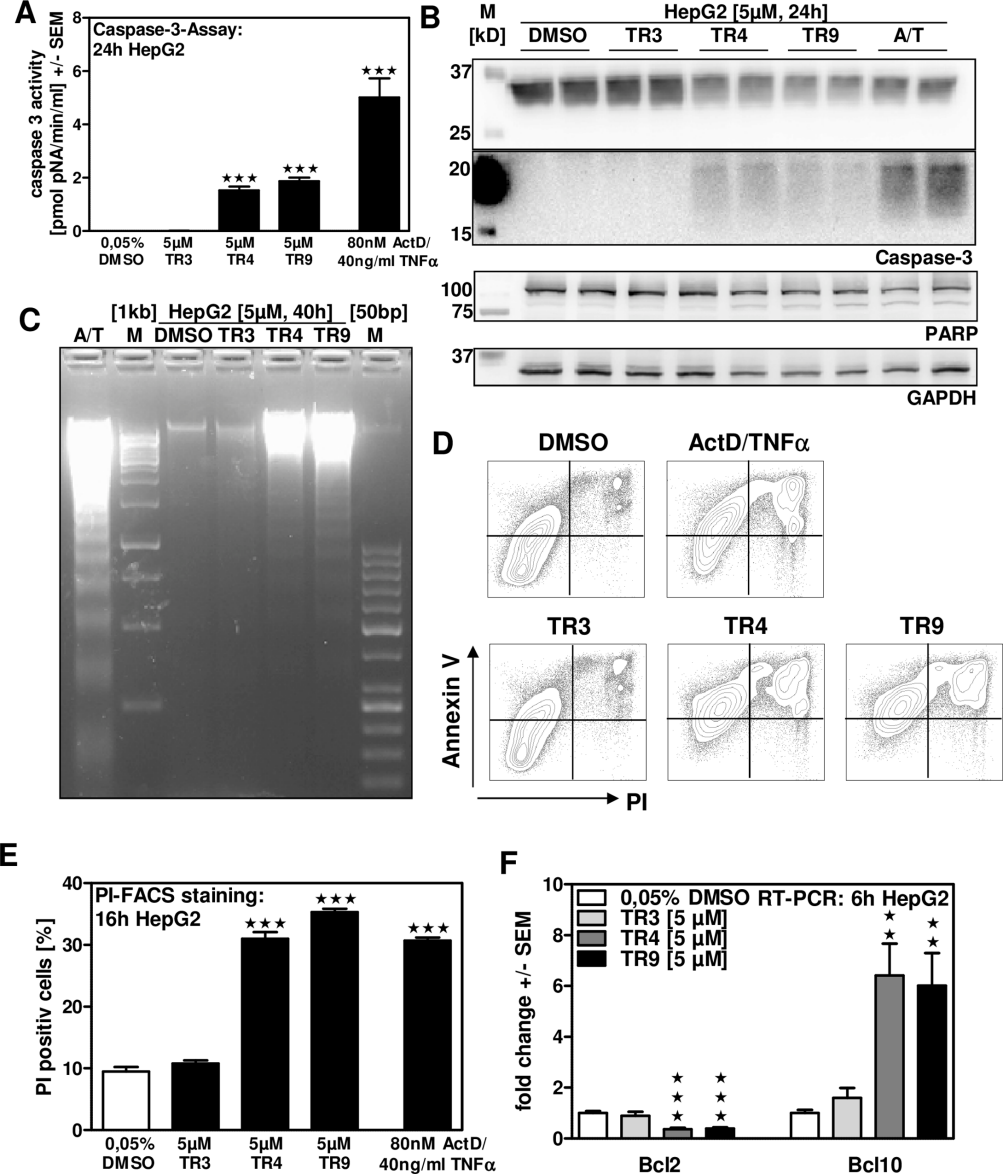
TR4 and TR9 induce apoptosis in human hepatoma cells. HepG2 cells were incubated with 5 μM TR3, TR4 or TR9 for 24 hours. As an apoptosis induction control HepG2 cells were incubated with 80 nM ActD as well as 40 ng/ml TNFα (ActD/TNFα) for 16 hours. Apoptosis was measured by Caspase-3 colorimetric assay (A) or by Western Blot analysis of Caspase-3- and PARP-cleavage (B). HepG2 cells were incubated with 5 μM TR3, TR4 or TR9 for 40 hours or with ActD/TNFα for 16 hours. Apoptotic DNA Ladder was determined by performing DNA-fragmentation analysis (C). HepG2 cells were incubated with 5 μM TR3, TR4, TR9 or with ActD/TNFα for 16 hours (D, E). FACS analysis was performed to detect apoptotic cells stained with propidium iodide (PI) and Annexin V. Representative plots are shown (D). E: Quantification of PI-positive HepG2 cells. F: HepG2 cells were incubated with 5 μM TR3, TR4 or TR9 for 6 hours. Real time RT-PCR for Bcl2 and Bcl10 expression was performed. Relative expression levels were determined using mATPsy expression as a reference. ★★ p ≤ 0.01, ★★★ p ≤ 0.001 vs DMSO controls.

Induction of apoptosis is an important point in cancer therapy. We therefore analyzed programmed cell death in HepG2 cells following limonoid incubation. Our results show that, similar to ActinomycinD/TNFα (ActD/TNFα) incubation, TR4 and TR9 activated Caspase-3 cleavage (Fig 4A and 4B), followed by PARP cleavage (Fig 4B) as well as fragmentation of genomic DNA (Fig 4C), whereas TR3 did not show any of these effects. FACS analysis using Annexin V and propidium iodide (PI) revealed an increase in PI-positive cells following TR4, TR9 or ActD/TNFα incubation up to 35%, while DMSO or TR3 incubation induced about 10% PI positive cells (Fig 4D and 4E). Furthermore, TR4 and TR9 reduced mRNA expression of anti-apoptotic Bcl2 and increase expression pro-apoptotic Bcl10 (Fig 4F). TR3 did not significantly affect expression levels of Bcl2 or Bcl10. Our results indicated that TR4 and TR9 induce apoptosis in hepatoma cells.

### TR4 and TR9 interfere with NF-κB signaling

**Fig 5:**
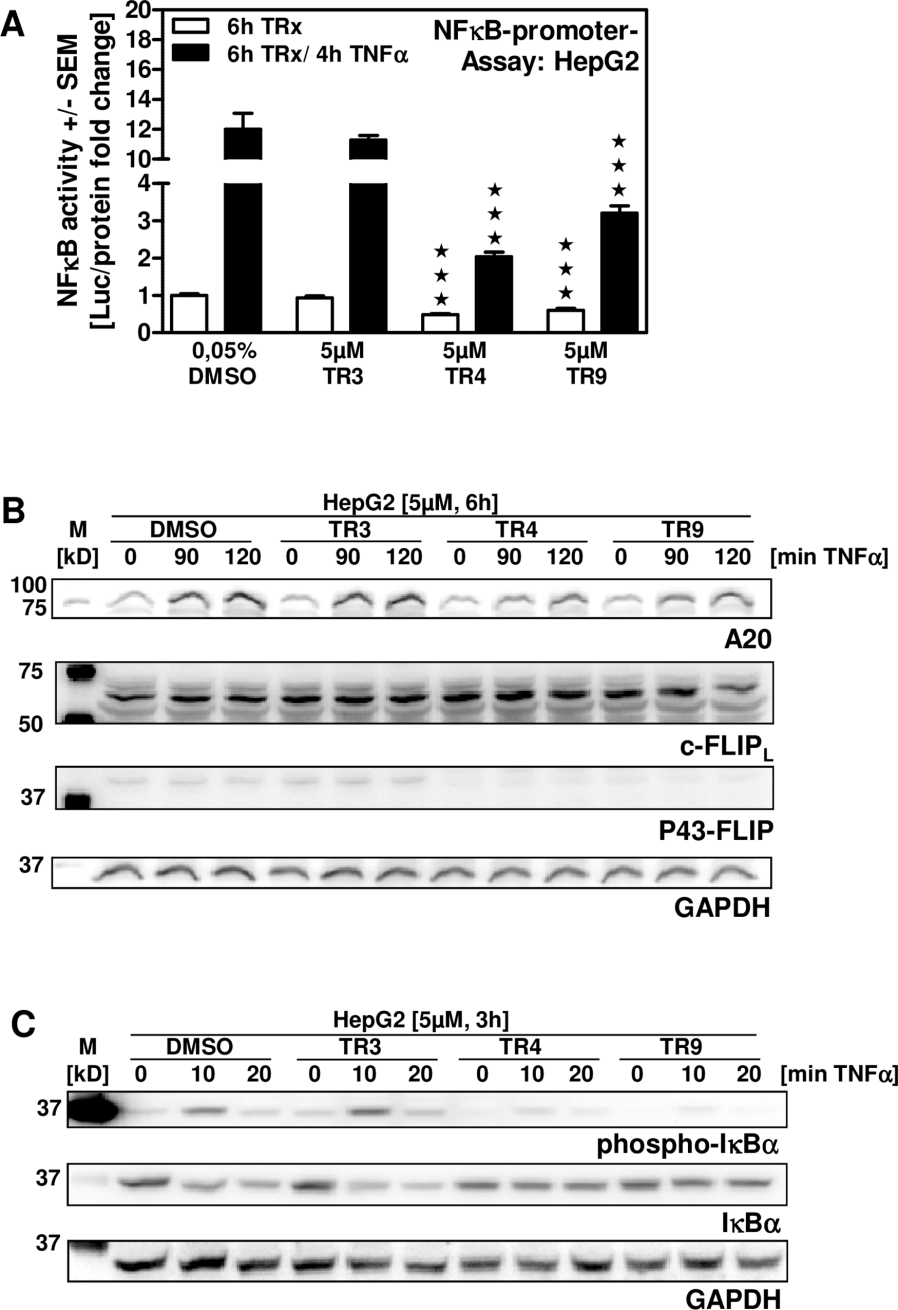
TR4 and TR9 interfere with NF-κB signaling. HepG2 cells were transfected with the NF-κB reporter vector pB2LUC for 24 hours. The next day cells were either incubated with 5 μM TR3, TR4 or TR9 for 6 hours (A, white bars) or pre-incubated with limonoids for 2 hours followed by TNFα incubation for 4 hours (A, black bars). NF-κB activity was determined by Luciferase Reporter Assay and quantified in relation to the protein content in the individual sample. (B) HepG2 cells were pre-treated with 5 μM TR3, TR4 or TR9 for 4 hours followed by TNFα incubation for 90 or 120 min. Protein expression of the NF-κB target gene A20, c- FLIP_L_ or p43-FLIP was detected by Western Blot analysis. (C) HepG2 cells were pre-treated with 5 μM TR3, TR4 or TR9 for 3 hours followed by TNFα incubation for 10 or 20 min. Phosphorylation and degradation of IκBα were measured by Western Blot analysis. ★★★ p ≤ 0.001 vs DMSO controls.

In cancers activity of the NF-κB signaling pathway is often found increased, resulting in cancer cell proliferation and survival [35]. Concomitantly, interference within this pathway induces cancer cell apoptosis. Our results show that TR4 and TR9 decreased NF-κB promoter activity in HepG2 cells, while TR3 did not (Fig 5A; white bars). In parallel experimental groups, NF-κB promoter activity was further increased by incubation with TNFα. Pre-treatment of cells with DMSO or TR3 followed by TNFα incubation increased NF-κB promoter activity about 5 fold higher than incubation with TR4 or TR9 in combination with TNFα (Fig 5A; black bars), showing that TR4 and TR9 both interfered with NF-κB activation. In addition we analyzed protein expression of the NF-κB target gene A20. Our results show increased A20 expression in HepG2 cells pre-treated with DMSO or TR3 followed by TNFα incubation, while the increase in A20 expression following pre-treatment with TR4 or TR9 was much less pronounced (Fig 5B, first line). An important step in NF-κB activation is the phosphorylation and degradation of the inhibitor of NF-κB IκBα. In DMSO or TR3 pre-treated cells we observed phosphorylation of IκBα within 10 min of TNFα incubation, leading to a rapid degradation of IκBα (Fig 5C). In contrast, we detected only slight amounts of phosphorylated IκBα as well as no degradation of IκBα in TR4 or TR9 pre-treated cells. In conclusion, TR4 and TR9 prevent degradation of IκBα and hence inhibit NF-κB signaling.

To confirm the inhibition of NF-κB we investigated the synthesis of an additional target gene, cellular FLICE like inhibitory Protein (c-FLIP). C-FLIP, also known as Caspase-8 and FADD like Apoptosis Regulator (CFLAR), binds at the death receptor next to pro-caspase-8. Hence, c-FLIP prevents binding of a second pro-caspase-8, the processing and activation of procaspase-8 and subsequently apoptosis [36].

C-FLIP is known to be up-regulated via several transcription factors including NF-κB [36]. Moreover, FLIP is known to be induced in various cancer cell lines and cancer tissue [36]. We measured protein levels of c-FLIP. Our results showed that the long form of c-FLIP (c-FLIP_L_) is highly expressed in the human hepatoma cell line HepG2 independently of TNFα stimulation (Fig 5B, second line). Furthermore, we analyzed the synthesis of p43-FLIP. p43-FLIP is generated upon caspase-8-mediated cleavage of c-FLIP_L_ providing that c-FLIP_L_ binds at the death receptor instead of the second pro-caspase-8 molecule [37]. In the pro-caspase-8/c-FLIP_L_ complex, caspase-8 is activated but cannot execute apoptosis [36]. Hence, formation of p43-FLIP indicates survival. Our results revealed p43-FLIP formation in solvent or TR3 treated cells but not in TR4 or TR9 treated cells (Fig 5B, third line). Therefore, we conclude that c-FLIP is executing its protective function in solvent or TR3 treated cells, but not in TR4 and TR9 treated cells.

### TR4 and TR9 support effects of Sorafenib on human hepatoma cell viability

**Fig 6:**
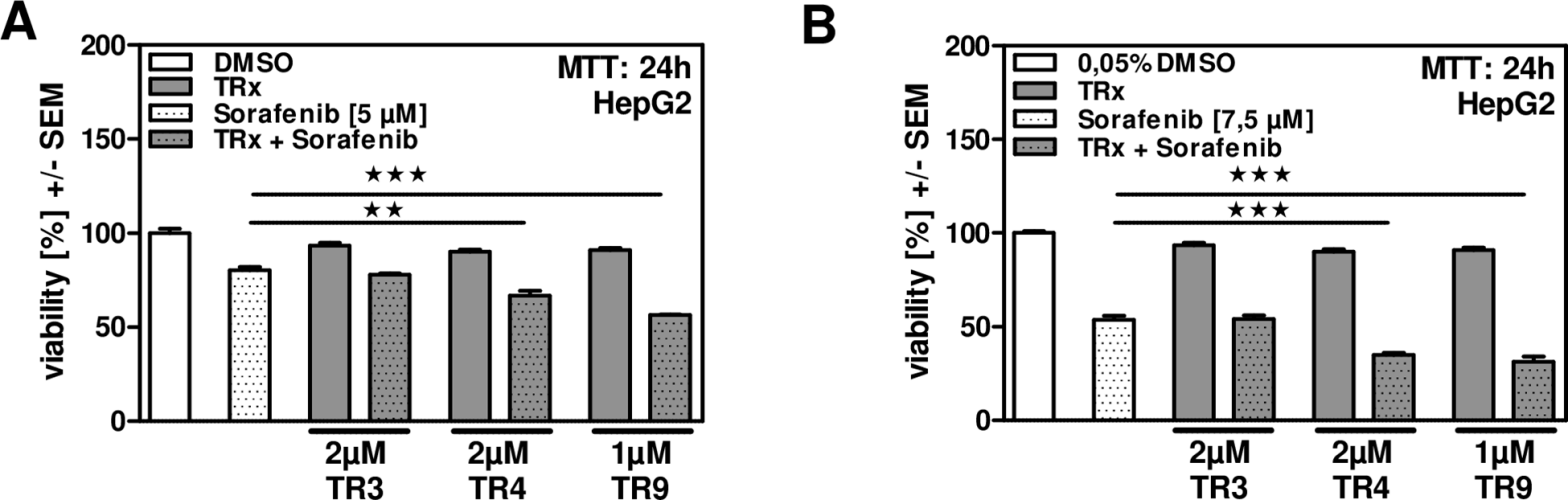
TR4 and TR9 support effects of Sorafenib on human hepatoma cell viability. HepG2 cells were incubated with 2 μM TR3, TR4 or 1 μM TR9 either alone or in combination with Sorafenib (A, 5 μM; B, 7.5 μM) for 24 hours. Cell viability was measured by MTT assay. ★★ p ≤ 0.01; ★★★ p ≤ 0.001.

To date use of the multi-kinase inhibitor Sorafenib is the most effective HCC treatment option in patients [7, 8]. We now investigated if a combination of Sorafenib with TR4 or TR9 might improve anti-cancer effects. Our results show that at a concentration of 5 μM Sorafenib alone reduced cell viability of HepG2 cells about 20% (Fig 6A). While combination of Sorafenib and TR3 did not further decrease cell viability, a combination therapy of Sorafenib and TR4 reduced cell viability by about 35%, while the combination of Sorafenib with TR9 reduced cell viability by about 45%. By increasing the concentration of Sorafenib to 7.5 μM we determined approximately 55% of viable cells (Fig 6B). Combination of Sorafenib and TR4 reduced cell viability by about 65%. By combining Sorafenib and TR9 we observed a reduction of cell viability by 70%. TR3 did not show any adverse effects on cell vitality in combination with Sorafenib. These results showed that TR4 and TR9 increase effects on cell viability in combination therapy approaches with Sorafenib.

## Discussion

For centuries, medicine relied on empirically discovered benefits of traditional medicinal plants without actual knowledge of the active compound or pharmacodynamics. Also, since the discovery of medical effects was empirical, many benefits of plant ingredients remained unknown. In our modern world, traditional medicine turned out to be a valuable source of knowledge und unexplored pharmacologically active substances [38].

Cancer is a major burden on public health. For many kinds of cancer, e.g. HCC, no satisfactory treatment is available. Substances derived from traditional medicine are under investigation for cancer treatment. Among those are animal products, e.g. bufalin, derived from toad’s skin or bee products[39]. Bufalin has been shown to induce cell cycle arrest and autophagy in human hepatoma cells in response to AKT/mTOR pathway activation [40]. In most studies however, plant products have been used [41–43]. For example isoliquiritigenin (ISL), a flavonoid found e.g. in licorice and different Radix spec., has been shown to induce apoptosis, e.g. in breast [44] and cervical cancer [45].

Based on the fact that, traditional African medicinal plants may contain naturally occurring compounds that effectively and specifically induce cell death in cancer cells, the objective for the present study was to evaluate the anticancer properties of some limonoids derived from Meliaceae, and especially from *Trichilia rubescens*. These substances might then represent a starting point for the development of new therapeutic treatments for cancer that do not respond to conventional chemotherapy regimens. Several species of the Meliaceae have a history of use in traditional medicine for the treatment of cancer. The wide occurrence of the limonoids in Meliaceae has evoked considerable interest in their anticancer activity. Limonoid are potentially cytotoxic substances and have been shown to exert anti-cancer effects in e.g. colon [13–15], breast [16–18] and hepatoma cell lines [19]. We investigated anti-cancer activities of 6 isolated and structurally characterized limonoids isolated from the African traditional medical plant *Trichilia rubescens* in human hepatoma cell lines. In fact, five out of these substances, TR4, 8, 9 11 and 12, significantly reduced cell viability while the sixth substance, TR3, did not. Comparing structures of the 2 most active substances, TR4 and TR9, to the inactive substance TR3 revealed close relationship with the exception of an epoxide ring structure present in TR4 and TR9, but not in TR3. In a parallel study, TR4, TR8, TR9, TR11 and TR12 were also evaluated for their anti-plasmodial activity against the 3D7 strain of *Plasmodium falciparum* [28]. The results obtained there indicate that the anti-malarial activity increased with the presence of epoxide rings in positions C-9/C-11 and C-14/C-15 in rings C and D, respectively. We therefore speculate that the epoxide ring structure is of eminent importance for the anti-cancer effects of TR4 and TR9. This is supported by the observation that Withaferin A, a substance with epoxide group in positions 5 and 6 possesses anti-cancer activity. The anti-cancer activity was significantly reduced by conversion of the epoxide groups into others chemical functions, confirming that the epoxide group can be linked to anti-cancer activity [46].

In order to evaluate if our compounds could be of significance for therapy, we compared TC50 values to those of drugs already established for cancer treatment. The anti-metabolite 5-FU is used in chemotherapy since the 1950s, with only limited success in hepatic cancer [47, 48]. Suberoylanilide hydroxamic acid (SAHA; Vorinostat) inhibits histone deacetylases type I and II, leading to apoptosis and anti-angiogenesis [49, 50]. SAHA was first used in therapy of cutaneous T-cell lymphoma [51]. Further studies revealed anti-proliferative effects e.g. on hepatoma cells [52] and in *in vivo* HCC models [53]. The multi-kinase inhibitor Sorafenib so far is the only approved HCC therapeutic which is able to prolong patient survival [7, 8]. Our results showed that TC50 values of TR4 and TR9 in HepG2 and Huh7 turned out to be much lower than TC50 values of 5-FU or SAHA. We also found that effects of 5 μM TR4 and TR9 were comparable to 10 μM of Sorafenib, indicating that TR4 and TR9 could become more potent anti-HCC drugs than Sorafenib. It is also notable that effects of TR4 and TR9 increased dose- and time-dependently up to at least 72 hours.

In this study we show for the first time that the two limonoids TR4 and TR9 initiate interference with hepatoma cell growth by compromising cell cycle progression and NF-κB pathway activation, resulting in induction of apoptosis.

Overexpression of proteins involved in cell cycle progression, e.g. Cyclin D1 in HCC patients and hepatoma cell lines, has been found to be associated with cancer development and progression [54, 55]. Anti-proliferative and pro-apoptotic properties have been reported for various natural compounds [13, 40, 44, 45]. In this study we show that TR4 and TR9 inhibit proliferation by down-regulating Cyclin D1 and PCNA as well as up-regulating p21, thereby interfering with cell cycle progression. Inhibition of cancer cell proliferation by reduction of Cyclin D1 expression and up-regulation of p21, an inhibitor of cell cycle progression, has been shown e.g. for the structural related triterpenoid nomilin [56]. Moreover, TR4 and TR9 induced multiple molecular characteristics of apoptosis in hepatoma cells, including caspase-3 activation, PARP cleavage, and significantly increased numbers of AnnexinV/PI positive cells as well as DNA fragmentation. Additionally, we observed decreased expression of the anti-apoptotic protein Bcl2, and increased expression of the pro-apoptotic protein Bcl10. Bcl2 has been shown to prevent apoptosis by e.g. binding to Bax and Bak, thus repressing caspase activation [57]. Expression of Bcl2 and anti-apoptotic Bcl2 homologues is controlled by NF-κB [35, 58]. It is well known that the NF-κB signaling pathway is constitutively active in HCC and promotes hepatocyte survival and proliferation [35, 59]. Many natural products, including some limonoids, exhibit their anti-cancer activity by interfering with the NF-κB signaling pathway. For example nimbolide has been shown to attenuate NF-κB signaling by inhibition of IKK activation in various cancer cell lines including leukemia, breast and kidney cells [60]. We therefore investigated the ability of TR4 and TR9 to interfere with the NF-κB signaling pathway. In fact we found significantly decreased NF-κB promoter activity in hepatoma cells in the presence of TR4 and TR9. This was due to diminished phosphorylation and subsequent degradation of the inhibitor of NF-κB, IκBα, leading to retention of NF-κB in the cytosol. It is not yet clear if TR4 and/or TR9 directly interfere with IκBα phosphorylation or if these limonoids modulate up-stream events in the NF-κB signaling pathway. This might be possible since Gupta and coworkers have shown that nimbolide modified Cys179 of IKK-β, a subunit of the IκB kinase complex [60]. Although the mechanism of how these limonoids interfere with the NF-κB signaling pathway has to be investigated in more detail, our results indicate that TR4 and TR9 reduce NF-κB activity in hepatoma cells, resulting in decreased expression of NF-κB targets (Cyclin D1, Bcl2 or A20). C-FLIP was not regulated in HepG2 cells, since these cells, as many other tumor cells, constitutively expressed c-FLIP [36]. However, lack of cleavage of c-FLIP_L_ to p43-FLIP clearly demonstrated that TR4 and TR9 induced cell death rather than survival [36]. The interference with the anti-apoptotic c-FLIP_L_ activity by TR4 and TR9 indicates that these limonoids stimulate an additional molecular mechanism by which they mediate their anti-cancer activity.

Addressing the question of drug safety, we switched to the mouse system. The advantage is that primary hepatocytes and hepatoma cells of the same genetic background can be compared (here: hepatocytes isolated from C57Bl6 mice vs. Hepa1-6 hepatoma cells). Biological activity of our compounds in mouse hepatoma cells was comparable to effects observed in human hepatoma cells, while we found no adverse effects of TR4 on viability of primary hepatocytes. TR9 reduced hepatocyte viability by about 20% compared to 60% reduction of viability in hepatoma cell. Although these results were obtained in the mouse system, they indicate that TR4 and TR9 affect tumor cells to a much larger extent than primary cells.

In summary, the natural compounds TR4 and TR9 induce apoptosis in hepatoma cells with a high selectivity compared to hepatocytes and a higher potency compared to Sorafenib. The mechanism involves inhibition of NF-κB activation, interference with cell cycle progression and subsequently induction of apoptosis. These are promising results, suggesting future use of TR4 and TR9, or derivatives, in HCC therapy.

## Abbreviations

HCC: hepatocellular carcinoma
MeOH: methanol
EtOAc: ethyl acetate
TMS: Tetramethylsilane
5-FU: 5-Fluorouracil
SAHA: Suberoylanilide hydroxamic acid
ActD: Actinomycin D
CCND1: Cyclin D1
ISL: isoliquiritigenin
TR4: TS3
TR9: Rubescin E
